# 566 Burn Disparities: Do Race, Gender and Insurance Status Affect Mortality?

**DOI:** 10.1093/jbcr/irac012.194

**Published:** 2022-03-23

**Authors:** Shevonne Satahoo, Juvonda Hodge, Victor C Joe

**Affiliations:** University of Miami, Miami, Florida; Emory University, Atlanta, Georgia; UCI Health Regional Burn Center, Orange, California

## Abstract

**Introduction:**

The National Inpatient Sample (NIS) provides ideas of trends across the country. A recent study in trauma patients found that there were no differences in race or insurance status in regard to mortality. As such, we sought to examine these effects in burn patients and hypothesized that these trends would be similar.

**Methods:**

The NIS was queried for all patients age ≥18 years with ICD-9 codes for total body surface area (TBSA) burn ≥ 20% and non-elective admissions. Years included were 2013 to the third quarter of 2015. Age, race, insurance status, TBSA, median household income for patient's zip code quartile and mortality were recorded. Cases with missing data for race, insurance status and mortality were excluded. Statistical analysis was done with student’s t-test and Chi-Square testing, as appropriate. Mortality was then used to run a binary logistic regression using these variables. A p-valve ≤0.05 was considered significant.

**Results:**

There were 5685 weighted cases. Females encompassed 28.3%. The mortality rate was 21.1%. The patients that died were older (58.6 ± 19.1 years versus 44.7 ± 17.1 years for survivors, p< 0.001).

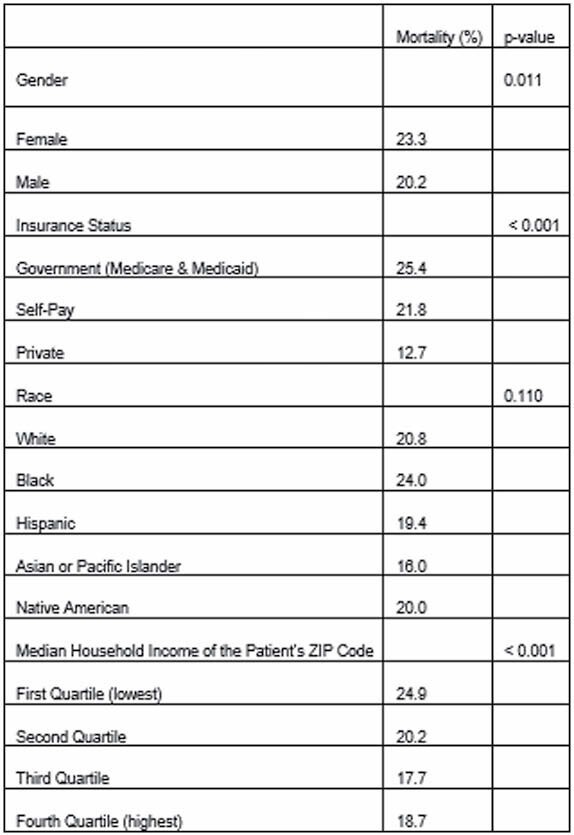

On multi-variate analysis of mortality using these variables, older age (p< 0.001), female gender (odds ratio (OR) 1.26 [1.05-1.50], p=0.011), lower median household income zip code quartile (highest quartile compared to lowest OR 0.69 [0.51-0.80], p< 0.001) and insurance status (compared to government insurance, private insurance OR 0.59 [0.47-0.72]; self pay OR 1.51 [1.18-1.93], p< 0.001) were associated with mortality. Increasing TBSA was also associated with mortality. Race was not a significant contributor (p=0.432).

**Conclusions:**

While there was a trend towards a higher rate of mortality in the black population, race was not associated with mortality in a statistically significant manner. Socioeconomic factors were associated with higher mortality. The dynamics between race and

other social determinants of health, and the potential impact of structural racism and bias should be the focus of future research rather than on race itself, especially considering that access and resources vary by state.

